# Disability among Palestinian elderly in the occupied Palestinian territory (oPt): prevalence and associated factors

**DOI:** 10.1186/s12889-019-6758-5

**Published:** 2019-04-25

**Authors:** Nouh Harsha, Luay Ziq, Rita Giacaman

**Affiliations:** 1 Faculty of Public Health, University of Debrecen, Debrecen, Hungary; 2 Institute of Community and Public Health, Birzeit University- ICPH/BZU, Birzeit, Occupied Palestinian Territory; 3 MPH, ICPH/BZU, UNRWA, Ramallah, West Bank, Occupied Palestinian Territory; 4 ICPH/BZU, Birzeit, Occupied Palestinian Territory

**Keywords:** Disability prevalence, Palestinian elderly, Age, Gender, Labor status, Palestinian refugees, Occupied Palestinian territory, West Bank and Gaza strip

## Abstract

**Background:**

Disability poses an important challenge to countries all over the world since it affects more than 15% of the global population. The disability prevalence is higher in developing countries compared to developed ones. Disability has negative consequences on health, wellbeing, and quality of life. The goal of this study is to assess the prevalence of disability and to determine some of its associated factors among Palestinian elderly in the occupied Palestinian territory (oPt), a country marked by a chronic lack of political, economic, and social stability which affect various aspects of the population’s life.

**Methods:**

We used data from the Palestinian Central Bureau of Statistics (PCBS) disability survey conducted in 2011 using a nationally representative sample of the Palestinians living in the West Bank (WB) and Gaza Strip (GS). Data were collected using a standardized questionnaire developed and adopted by the World Health Organization (WHO) and the Washington Group (WG) for Disability Statistics, adapted to satisfy the Palestinian context.

**Results:**

Overall, 31.2% of the Palestinian elderly 60 years and above reported one or more type of disability. Binary logistic regression with disability as the dependent variable showed that older people [OR = 2.88, 95% CI: 2.31–3.60], women [OR = 1.65, 95% CI: 1.33–2.04], illiterate people [OR = 2.37, 95% CI: 1.83–3.06], people reporting small family sizes with 1 to 2 members [OR = 1.69, 95% CI: 1.34–2.14], people who reported that they were not working at the time of the survey [OR = 4.59, 95% CI: 3.13–6.73], and Palestinian refugees [OR = 1.22, 95% CI: 1.04–1.42] were more likely to have a disability. However, residents of the Centre of WB were less likely to have disability compared to residents of the GS [OR = 0.46, 95% CI: 0.37–0.58].

**Conclusions:**

The study found a high prevalence of disability among Palestinian elderly, as has been reported by the majority of studies performed in developing countries. However, results indicate that demographic and socioeconomic differences among the disabled should be taken into special consideration in setting policies and practices to improve the health and wellbeing of the disabled.

## Background

In 2006, the Convention on the Rights of Persons with Disabilities (CRPD) broadly defined the disabled as “those who have long-term physical, mental, intellectual or sensory impairments which in interaction with various barriers may hinder their full and effective participation in society on an equal basis with others” [1]. Disability has become a public health concern in recent decades [2, 3]. The universal increase in life expectancy with a consequent rise in the number of the elderly population around the world is resulting in an increase in the prevalence of disability [4]. For instance, in Japan, the elderly aged 65 years and more constituted 23% of the population in 2010 [5]. In addition, the increasing ill health experiences which come with age, including acute and chronic conditions, injuries and accumulation of their effects over the life course enhance the rates of disability [6].

Disability poses an important challenge to the world’s population since it affects more than one billion (15% of the world population based on 2010 estimations) persons around the globe, a prevalence which is higher in the developing countries compared to the developed ones [7]. Indeed, a World Health Survey on 53,447 people aged 50 years and above conducted in 43 low and middle-income countries reported an overall disability prevalence of 33.3% [8]. In Japan, a cross-sectional study completed on 1550 participants aged 65 years and over showed that the percentage of functional disability -defined as limitations occurring over long period of time due to illness, condition, or an injury [9]- was 20.1% [4]. Another study conducted in Malaysia in 2015 to determine prevalence and determinants of disability among adults using a national health and mobility survey, reported that of those aged 61 years and above, 41.0% had one or more types of disability [10]. A study conducted in rural Haryana of India to assess functional disability among 836 elderly aged 60 years and over reported that the prevalence of functional disability was 37.4% [11].

Disability has negative consequences on health and quality of life [6]. Studying disability is essential to understand and manage the health of the elderly since the majority of them have several comorbidities with a serious impact on daily activities, health, and wellbeing [12]. In fact, physical disability associated with a range of diseases has been shown to be a good predictor of mortality among the elderly [13]. In addition, disability reduces people’s chances for a good education, job, and income, putting them at higher risk of poverty, low socioeconomic status, poor housing conditions and low access to nutritious food and health care services, and hence, worse health conditions and increased dependence on others [7, 14, 15]. Disability in later life was reported to increase elderly social exclusion and depression [16, 17]. Disability also increases the costs of health care. In 2006, the disability cost was estimated to account for 26.7% of the total health care cost for adults in the United States [18].

The prevalence of disability is likely affected by several factors. The main factors identified in the literature were demographic characteristics and socioeconomic factors including age, sex, race, education, and marital status [8, 12], income status and occupation [19], and living alone [6]. A study conducted in Thailand to assess factors associated with the six types of disability (seeing, hearing, mobility, remembering and concentrating, communication, and personal care) for people 60 years old and above reported that the presence of other comorbidities was found to increase disability prevalence [20]. Furthermore, psychosocial factors (such as social support) have an influence on health, chronic conditions, functional limitations and disability [21, 22]. In 2014, a study completed in Bangladesh to assess the association between disability and wealth concluded that with increasing wealth there was a linear decrease in the probability of having a disability [23]. In addition, disability prevalence was found to vary by place of residence in urban or rural areas, and to the geographical areas or region [24].

A move away from the medical model to the social model for understanding and managing disability has taken place during the past few decades [19]. Although health conditions play a major role in the occurrence and distribution of functional limitations and disability, it is reported that physical, social, economic and environmental conditions are major determinants of individuals functioning, participation, and involvement in the society [3, 25]. Due to the increase in the average life expectancy of the population in developing countries leading to a higher proportion of elderly population [26, 27], disability among the elderly has attracted researchers and policy makers attention [28]. In the occupied Palestinian territory (oPt), life expectancy has increased by 5 to 8 years, rising from 67.0 years in 1992 to 72.3 years and 75.4 years in 2017 for men and women respectively [29]. However, the lack of social, economic and political stability in the oPt [30] are factors reported to have a serious negative impact on the disabled people and their families [7].

Since disability affects both morbidity and mortality of the population and given that it is essential to understand and manage population aging [12], studying disability in the oPt is necessary especially in view of the particular difficult political conditions in which Palestinians live and the rising average life expectancy. Disability in the Palestinian context has not been well studied particularly among the elderly. This is why this study deals with disability among elderly Palestinians living in both the West Bank (WB) and the Gaza Strip (GS). The goal of this study is to estimate the prevalence of disability among Palestinian elderly aged 60 years and above and to determine some of its associated factors. We hypothesize that the prevalence of disability among Palestinian elderly aged 60 years and above is high and comparable to other developing countries.

## Methods

### Data source

We used data from The Palestinian Central Bureau of Statistics’ (PCBS) Disability National Survey conducted in 2011 in collaboration with the Ministry of Social Affairs, using a representative sample of Palestinians living in the WB and GS calculated based on the Population Housing and Establishment Census of 2007. The goals of this survey were to measure prevalence of disability types, the ability of the disabled to cope with the surrounding environment, to participate in work, education, and social activities, and to identify their needs as well as the services provided to them.

The sample covered the 16 governorates of the oPt of all locality types: Urban, rural, and camps. Initially, the area was stratified by governorate and locality type. Each locality was divided into a number of enumeration areas based on the population size to ensure fair representation of all the areas. Each enumeration area consisted of 120 housing units. Sample design involved two stages. In the first stage, a stratified random sample of 314 enumeration areas was selected (211 in the WB, and 103 in the GS). In the second stage, 50 households were selected from each numeration area selected in the first stage. Data was collected using a standard questionnaire adopted by the World Health Organization (WHO) and the Washington Group (WG) for Disability Statistics, taking in consideration the Palestinian context, the international recommendations related to disability and feedbacks from local experts in the disability field for the benefits of the survey. Two questionnaires were designed: One for children aged 0–17 years, and another for adults aged 18 years and above. The sample size was 15,572 households. Data collection was completed during January and March of 2011 [31].

### Outcomes measured

The study’s outcome was disability among elderly Palestinians aged 60 years and above. It was calculated in line with the recommendations of WG for Disability Statistics [32], by building a scale consisting of various forms of reported disability. Five main types of disabilities were used: Seeing, hearing, mobility, remembering and concentrating, and communication disabilities covering intellectual, psychological and mental health disabilities. The disability domain related to personal care which is the sixth domain in WG for Disability Statistics was not included in data analysis since the majority of the cases were missing from the data set. Mobility disability was determined using five questions related to having disability/difficulty in moving inside the home, outside the home, moving for 15 min or longer, use of hands and fingers and ability to raise two liters of water (Appendix 1). Remembering and concentrating difficulty was determined with reports of having difficulty in remembering to do an important thing, forgetting where you have put things and difficulty in concentrating on doing something for 10 min (Appendix 2). Responses to this domain were obtained from either the head of the household or a qualified household member who knows the situation of the disabled well after making sure that either of them can provide accurate information about the disabled as has been demonstrated by a pilot study conducted prior to the main survey. Communication disability was determined by having difficulty in intellectual function due to a health condition, autistic disorder, or learning daily skills (Appendix 3). A 5-point Likert scale (1 = no difficulty, 2 = some difficulties, 3 = a lot of difficulties, 4 = cannot at all, and 5 = do not know) was used. Do not know responses were reported by 9 respondents in response to their intellectual functions, and they were excluded from the final data analysis. The scale was recoded into 4 categories from 0 (no difficulty) to 3 (cannot at all). Disability which is the dependent variable was defined as experiencing a lot of difficulties or cannot at all on any single disability domain, or having some difficulties on at least 2 domains. This definition has been reported in several studies [10, 33, 34].

### Independent variables

Independent variables included gender, age groups: 60–69, 70–79, and 80 years and above. Educational levels were categorized as: Illiterate for those who did not received any formal education, less than secondary education, and those with secondary education and above. Marital status included married, widowed, and others including singles, engaged, divorced and separated. Family size refers to the number of persons living in the same residential unit and whether living in nuclear or extended families. Labor status included: employed people for those working one to 35 h a week to gain profits or wages, house workers for those involved entirely in the household chores without gaining wages, and not working for all other categories. Refugee status and region included North WB, Center WB, South WB, and the Gaza Strip.

### Statistical analysis

We calculated the overall prevalence of disability among elderly Palestinians aged 60 years and above, the dependent variable for this study. We performed univariate analysis for all independent variables to identify basic study sample characteristics. Bivariate analysis was carried out using Chi-square to assess the association between the study outcome and various independent variables. Finally, to determine the predictors of disabilities in the oPt, multivariable logistic regression was performed. The variables included in the model were Age, sex, education, marital status, family size, nuclear or extended families, labor force, refugee status, and region. The level of significance was determined at ‘*p* < 0.05’. Data analysis was completed using SPSS® version 20. No inclusion and exclusion criteria were specified in the original survey. This study was conducted in line with the Palestinian General Statistical Law of 2000, which allows the PCBS to conduct national surveys and emphasizes voluntary participation, confidentiality, and protection of the individuals and the data [35].

## Results

Table 1 below contains the background characteristics: 56.4% of the respondents were 60 to 69 years old. Mean age was 69.1 for men and 70.3 for women. 55.8% were women, 17.2% had secondary or postsecondary education, 63.8% were married, 24.3% of the families included 7 members or more, 21.0% were extended families, 10.5% were employed, 40.9% were refugees, 33.2% of the respondents were resident in the north of the WB, 20.4% in the center of the WB, 18.0% in the south of the WB, and 28.4% in the GS.

**Table 1:** Background characteristics of elderly persons living with disability in the occupied Palestinian territory (oPt) (*N* = 4298)

Variable		Number	Percentage (%)
Age groups	60–69	2422	56.4
	70–79	1287	29.9
	80 and above	589	13.7
Gender	Male	1899	44.2
	Female	2399	55.8
Education	Illiterate	1868	43.5
	Less than secondary	1691	39.3
	Secondary and above	739	17.2
Marital status	Married	2742	63.8
	Widowed	1341	31.2
	Others	215	5.0
Family size (person)	1–2	1339	31.2
	3–6	1916	44.5
	7 and above	1043	24.3
Family structure	Nuclear	3394	79.0
	Extended	904	21.0
Labor force	Employed^a^	453	10.5
	House worker	1098	25.5
	Not working	2747	64.0
Refugee status	No	2541	59.1
	Yes	1757	40.9
Region	North WB^a^	1429	33.2
	Center WB	875	20.4
	South WB	775	18.0
	Gaza Strip	1219	28.4

^a^The Employed are those working one to 35 h a week to gain profits or wages, house workers are those involved entirely in the household chores and not gaining wages. *WB* West Bank

The prevalence of disability among people aged 60 years and above was 31.2% (Table 2). Among those aged 60–69 years, 21.1% were disabled compared to 56.7% of those aged 80 years and above (*P* < 0.05). Men had lower rates of disability at 24.2% compared to women at 36.7% (*P* < 0.05). Disability was higher among the illiterates at 42.5% compared 15.3% among those with secondary or post-secondary education (*P* < 0.05). Of the married, 24.7% were disabled compared to 43.7% of the widowed (*P* < 0.05). Family size was important, with 39.7% of the families with 1 to 2 members found disabled compared to 29.4% of those with 7 members or more *P* < 0.05). Those living in nuclear families had lower rates of disability at 29.1% compared to 39.0% of those living in extended families (*P* < 0.05). Those employed had lower rates of disability at 7.1% compared to 38.5% of those who were unemployed at the time of the survey (*P* < 0.05). Non-refugees had lower rates of disability at 29.8% compared to 33.1% among the refugees (*P* < 0.05). Finally, 20.1% of the residents of the center of the WB were disabled compared to 32.7% of those living in the GS (*P* < 0.05).

**Table 2:** Disability prevalence by demographic and socioeconomic characteristics (*N* = 4298)

Variable (n)		Not disabled (%)	Disabled* (%)	Chi square*
Age groups	60–69 (2422)	1910 (78.9)	512 (21.1)	323.2
	70–79 (1287)	794 (61.7)	493 (38.3)	
	80 and above (589)	255 (43.3)	334 (56.7)	
Gender	Male (1899)	1440 (75.8)	459 (24.2)	77.4
	Female (2399)	1519 (63.3)	880 (36.7)	
Education	Illiterate (1868)	1075 (57.5)	793 (42.5)	222.1
	Less than secondary (1691)	1258 (74.4)	433 (25.6)	
	Secondary and above (739)	626 (84.7)	113 (15.3)	
Marital status	Married (2742)	2064 (75.3)	678 (24.7)	152.6
	Widowed (1341)	755 (56.3)	586 (43.7)	
	Others (215)	140 (65.1)	75 (34.9)	
Family size	1–2 persons (1339)	807 (60.3)	532 (39.7)	70.2
	3–6 persons(1916)	1416 (73.9)	500 (26.1)	
	7 and above (1043)	736 (70.6)	307 (29.4)	
Family structure	Nuclear (3394)	2408 (70.9)	986 (29.1)	33.3
	Extended (904)	551 (61.0)	353 (39.0)	
Labor force	Employed (453)	421 (92.9)	32 (7.1)	228.7
	House worker (1098)	849 (77.3)	249 (22.7)	
	Not working (2747)	1689 (61.5)	1058 (38.5)	
Refugee status	No (2541)	1784 (70.2)	757 (29.8)	5.4
	Yes (1757)	1175 (66.9)	582 (33.1)	
Region	North WB (1429)	913 (63.9)	516 (36.1)	67.8
	Center WB (875)	699 (79.9)	176 (20.1)	
	South WB (775)	527 (68.0)	248 (32.0)	
	Gaza Strip (1219)	820 (67.3)	399 (32.7)	
Total (4298)		2959 (68.8)	1339 (31.2)	

*The disabled are those who reported a lot of difficulties or cannot at all on any single domain, or those who reported having some difficulties on at least 2 domains. *P value was <0.001 for all the variables presented in the table except for refugee status were *P* value was 0.011

Table 3 represents results of the multivariate logistic regression for disability. People aged 80 years and above were around 3 times more likely to have a disability compared to people aged 60 to 69 [OR = 2.88, 95% CI: 2.31–3.60]. Women were about two times more likely to have a disability compared to men [OR = 1.65, 95% CI: 1.33–2.04]. Illiterate were about two times more likely to have a disability compared to people with secondary and post-secondary education [OR = 2.37, 95% CI: 1.83–3.06]. Families with 1 to 2 members were about twice more likely to have a disability compared to families with 7 members or more [OR = 1.69, 95% CI: 1.34–2.14]. People who were not working were about five times more likely to have a disability compared to those working [OR = 4.59, 95% CI: 3.13–6.73]. Refugees were more likely to have a disability compared to non-refugees [OR = 1.22, 95% CI: 1.04–1.42]. Residents of the center of the WB were about twice less likely to have a disability compared to residents of GS [OR = 0.46, 95% CI: 0.37–0.58].

**Table 3:** Predictors of disability among Palestinian elderly in the oPt

Variable		Unadjusted OR^a^ (95%C.I.)	Adjusted OR^a^	95% C.I. for OR^a^		*P*-value
				Lower	Upper	
Age	60–69	1	1			
	70–79	2.32(2.00–2.69)	1.51	1.27	1.78	< 0.001
	80 and above	4.89(4.04–5.91	2.88	2.31	3.60	
Gender	Male	1	1			
	Female	1.82(1.59–2.08)	1.65	1.33	2.04	< 0.001
Education	Secondary and above	1	1			
	Illiterate	4.09(3.28–5.09)	2.37	1.83	3.06	< 0.001
	Less than secondary	1.91(1.52–2.40)	1.81	1.42	2.30	
Family size	7 and above	1	1			
	1–2 persons	1.58(1.33–1.88)	1.69	1.34	2.14	< 0.001
	3–6 persons	0.85(0.72–1.00)	1.23	1.01	1.51	0.043
Labor status	Working	1	1			
	House worker	3.86(2.62–5.68)	1.83	1.21	2.79	0.004
	Not working	8.24(5.71–11.90	4.59	3.13	6.73	< 0.001
Refugee status	No	1	1			
	Yes	1.17(1.02–1.33)	1.22	1.04	1.42	0.012
Region	Gaza Strip	1	1			
	North WB	1.16(0.99–1.37)	1.06	0.88	1.28	0.514
	Center WB	0.52(0.42–0.64)	0.46	0.37	0.58	< 0.001
	South WB	0.97(0.80–1.17)	0.91	0.72	1.13	0.382

^a^The variables inserted and adjusted for were age, sex, education, marital status, family size, family structure, labor status, refugee status, and region, *C.I* confidence interval, *OR* odds ratios, *WB* West Bank

## Discussion

We investigated disability and some of its associated factors using a nationally representative sample of Palestinians elderly (60 years and above) living in the WB and the GS. According to the PCBS 2017 statistics, the elderly aged 60 years and above constituted 5.0% of the total Palestinians living in the oPt [29]. Overall, prevalence of disability in the oPt was 31.2% which is high and comparable to the high prevalence of disability among the elderly in developing countries [8, 11].

As age advances, people become more likely to have a disability. This finding corresponds to the results of Parker and colleagues who reported an increase in disability prevalence among the older elderly [36]. Indeed, age is regarded as one of the main predictors and determinants of elderly disability [37]. It is maintained that in Japan, the rate of disability is likely to be doubled with each 5 years age advancement [4]. This could be explained from a physiological point of view, since body organs become aged there is an increase in overall acute and chronic health conditions leading to functional limitations and disability [36, 38]. Older adults are usually disadvantaged and vulnerable and are unable to afford health care costs required for improving their health status and quality of life [6].

Women were more likely to have a disability compared to men. This finding was supported by several other findings reported in the literature [8, 36]. Various reasons were proposed to explain this difference. Some attributed this to the high rate of acute and chronic diseases and health problems among women who are more likely to report health illness and disability compared to men [39–41]. This could also be attributed to the higher life expectancy among women compared to men [40]. Furthermore, women as a disadvantaged group in society usually suffer from lack of education, unemployment, lower income, unhealthy food, poor working and living circumstances, and less access to health care services leading to a higher disability prevalence compared to men [13, 42, 43].

Educated people were less likely to have a disability. This finding coincides with findings from other studies which reported protective effects of education on the health status of the elderly [8, 23]. In 2011, Zitko Melo and colleagues reported that education affects lifestyle and helps in preventing ill health status in general [37]. In fact, education increases the personal knowledge and awareness and facilitate access to information deemed necessary for the wellbeing [6, 19]. In addition, highly educated people are more likely to adhere to rehabilitation programmes and thus have better health outcomes compared to less educated people [19]. Furthermore, educated people are more likely to get a job, improve their socioeconomic and financial position known well to positively enhance health care access and health status [6, 44].

Those who do not work were more likely to have a disability compared to working persons. This was a major finding. Indeed, several studies have reported that the disabled were less likely to be employed [6, 37]. Disability has been shown to reduce the chances of obtaining a job and enhancing poverty [14]. In fact, a number of studies reported that the disabled have low levels of education, thus more likely to have fewer chances of employment, and even if employed, they are more likely to earn a lower income than nondisabled people [7, 15, 45]. In addition, low levels of education leading to low income and low standards of living do not help people access proper healthcare services, good and healthy food and hinder them from looking after themselves [6, 42].

Elderly people living in small families with 1 or 2 members were more likely to have a disability compared to those living in families with 7 members or more. This result is consistent with the literature which revealed that majority of the care of the elderly is the responsibility of family members [46] which is also the case in the Palestinian society. The absence of family members, especially younger ones, reduces the care given to the elderly. Likewise, WHO reported that elderly people are at greater risk of falling into poverty when family size is reduced [47]. The trend towards decreasing fertility and the increase in nuclear family arrangements is likely to disrupt or compromise the capacity of the family to offer optimum personal and financial care to its elderly members [48, 49]. Furthermore, small families have less adult children creating fewer chances for offering both emotional and financial support for the elderly [50].

Refugees were more likely to have a disability compared to non-refugees. A similar finding was reported by Strong and colleagues in a study conducted among Syrian refugees in Lebanon who demonstrated high levels of chronic conditions and disability. These refugees included Syrian citizens and Palestinian refugees who were resident in Syria and who have become refugees for the second or even third time. Interestingly, Palestinian refugees were reported to have a two to five folds higher disability prevalence compared to Syrian refugees [51], perhaps because of having become refugees more than once. Elderly people are vulnerable, and living in refugee camp localities is associated with increasing possibilities of impairment and disability, a trend that can be attributed to poor health conditions, unhealthy food, poor housing conditions, unmet healthcare needs, injuries, accidents, trauma and torture [52].

Elderly people living in the GS were more likely to have a disability compared to those living in the center of the WB. Parker and colleagues studied elderly disability and physical illness in England and Wales and likewise reported a regional variation by levels of disability at the national level [36]. In 2014, a study conducted in the Flemish region of Belgium reported that disability was associated with poverty and low access to health care services [14]. In addition, education, employment, income and availability of health care services were reported to be major determinants of disability [6, 12, 23]. The differences noted in the oPt regions could be attributed to variation in economic, political and developmental factors. Those factors affect life and health of the elderly and their families and their way to have a good income, nutritious food, access to health care services [53]. Indeed, the choking siege of the Gaza Strip affecting all civilians has been associated with a low health related quality of life of all Gazans, including the elderly and the disabled elderly [54]. In contrast, the central West Bank district includes Ramallah city which is the seat of power for the Fatah-led Palestinian Authority, and is the headquarters for most international non-governmental organizations (NGOs) and embassies, where hundreds of millions of dollars in aid are poured. Even when compared to the other districts of the WB, Ramallah is known to have a stronger economy than the north or southern districts of the WB [55].

Strength of this study comes from the fact that a large nationally representative sample of the Palestinians living in the oPt was used thus data generated are valid and reliable. However, several limitations can be raised. This is a cross-sectional study which identifies association but not causation. In addition, we could not distinguish between people with preexisting disability before becoming elderly and those who became disabled during their elderly years. Furthermore, important possible predictors other than demographic and socioeconomic factors which could better explain the prevalence of disability were not included in the study. Those include, inter alia, political crisis, chronic illnesses, obesity, physical activities, and psychosocial factors.

## Conclusions

There is a high prevalence of disability among Palestinian elderly living in oPt as has been reported in the majority of the developing countries. We found that older people, women, illiterate people, people reporting living in small families sizes with 1 to 2 members, people who reported that they were not working at the time of survey, Palestinian refugees, and residents of the GS were more likely to have a disability. These factors need to be taken into consideration when developing policies and interventions geared towards supporting the elderly disabled and their families. In the oPt, successive and collective efforts were made by the Palestinian Union of Disabled People and local and international NGOs calling for a disability law, which culminated in the promulgation of this law by the Palestinian Legislative Council in 1999 [56]. However, the development of mechanisms for the implementation of this law with appropriate policies and interventions has yet to be made. We hope that the results of this study would be used as tools for policies and interventions geared towards fulfilling the needs of the Palestinian elderly in general, and not only the disabled elderly. Such needs should include not only medical and health care, but also social protection in the form of social security benefits which can allow the elderly to seek health and disability care, and live the last part of their life in dignity.

## Appendix 1

1. Moving inside home difficulty/disability.
2. Moving outside home difficulty/disability.
3. Moving for 15 min and longer difficulty/disability.
4. Use of hands and fingers difficulty/disability.
5. Ability to raise 2 l of water difficulty/disability.

## Appendix 2

1. Difficulty in remembering to do an important thing.
2. Forget where you have put things.
3. Difficulty concentrating on doing something for 10 min.

## Appendix 3

1. The difficulty with intellectual functions due to a health condition.
2. The difficulty with intellectual functions due to autistic disorder.
3. Difficulty in learning every skill (daily skills).

## Abbreviations

CI: Confidence interval
CRPD: Convention on the Rights of Persons with Disabilities
GS: Gaza Strip
NGOs: Non-governmental organizations
oPt: occupied Palestinian territory
OR: Odds ratio
PCBS: The Palestinian Central Bureau of Statistics
WB: West Bank
WG: Washington Group
WHO: World Health Organization
